# Tobacco Amblyopia

**Published:** 1910-04-23

**Authors:** Percy Dunn

**Affiliations:** Senior Ophthalmic Surgeon, West London Hospital; Lecturer on Ophthalmology, West London Post-Graduate Course.


### HOSPITAL CLINICS.

### TOBACCO AMBLYOPIA.

BY PERCY DUNN, F.R.C.S., Senior Ophthalmic Surgeon, West London Hospital; Lecturer on Ophthalmology, West London Post-Graduate Course.

To those unfamiliar with the subject of tobacco amblyopia the cases are somewhat puzzling because of the negative character of the symptoms. The eyes exhibit nothing abnormal externally, and with the ophthalmoscope no deviation is apparent from natural appearances. Yet still the patient complains of a serious declension of vision. He alleges great difficulty in seeing clearly distant objects: his work, whatever it may be, owing to his failure of sight, is becoming more and more difficult, and reading is only possible when the type is large. What, then, is the diagnosis? Rightly to determine this is of paramount importance in such cases, inasmuch as if the real cause of the loss of sight be overlooked, the patient runs the serious risk of passing into a condition of almost total blindness, which proves to be permanent. Let us see if any facts can help us towards arriving at a diagnosis, apart from examination of the eyes. I repeat apart from the examination of the eyes for, as you know, I am always accustomed to insist, in the examination of eye cases, upon the importance, the expediency, the usefulness of examining the eyes last. This may appear to be a paradoxical precept; on the contrary, in the ordinary routine of ophthalmic work its value is repeatedly proved. Remember then first in all cases to take note of everything apparent, apart from the eyes. Observe carefully the patient's face, noting the condition of the skin, the colour of the complexion, the physiognomy, and whether any scars or an eruption are present, or whether any scarring exists upon the sides of the neck. A glance will determine these various points. A man, for example, comes with one eye covered. One can guess what is probably the matter with it without looking, for his forehead is covered with a profuse papular syphilide. Again, a child is brought with acute inflammation of its corneæ: the diagnosis is shown on the face, for that displays the characteristic signs of congenital syphilis, a fact which the subsequent examination of the teeth confirms. So also it happens that the man who comes with tobacco amblyopia is not without certain features which at once raise suspicions respecting the source of his trouble. A typical case of the kind may be described as follows. The middle-aged patient walks boldly into the out-patient room, acts as if he saw plainly, and neither in gait nor in gesture displays the loss of vision which is present in his case. The burden of his complaint is that he is losing his sight. While making his statements, however, we notice the pallor of his face, the tremulousness of his hands, the furring on the dorsum of his tongue, as is apparent when he is told to show it. To test for the tremors direct the patient to stretch out his arms straight forward from the shoulders, the hands approximating, but not touching the thumbs. The eyes showing no signs of external disease, we next proceed to ask him some questions, from his replies to which we ascertain the following details. That everything for about the past three months has appeared to be "in a fog," as he expresses it; that he can only read large print; that the sight is now so bad that he has been compelled to cease his employment; that for some time he has felt "shaky"; that he has no appetite for his breakfast; that he has great difficulty in distinguishing between the colour of gold and silver; that he has sleepless nights; and that he has particularly noticed that everyone he meets looks ill, their faces being the colour of yellow wax. Next we inquire as to his habits. He admits that he smokes from a quarter of an ounce to half an ounce of shag daily; that he has done so for many years; that directly he rises in the morning he begins to smoke, and continues doing so, off and on, while at work, until his mid-day meal. Then his vision is examined. At the distant types the record is 6/nil, without any improvement. His near vision is also much reduced, amounting to J 16, less or more, at 8 to 9 inches, according to the case. Examination of the fundi with the ophthalmoscope, in the dark room, reveals nothing. In some cases there may be slight hyperæmia of the disc, in others temporal pallor, but in neither instances are the appearances typical. Finally, we proceed to the last test, by which practically is established the diagnosis of the case. This consists of an inquiry into the integrity of the colour vision, concerning which a few details may now be given. First it may be pointed out that the fields for colour perception vary in their size, in the natural eye. The smallest is that for green; the next in size is red, then yellow, and lastly blue, which is the largest. In examining these fields in cases of tobacco amblyopia we find a defect in those for red and green. This defect takes the form of a central scotoma, usually of an irregular oval in shape, lying horizontally between the blind spot and the macula. A ready and fairly satisfactory method of examining a patient for central scotoma in the absence of a perimeter is as follows: Place the patient in a chair with his back to the light. With the surgeon sitting opposite, the patient is directed to cover one eye with his hand, and with the other to look straight at the surgeon's nose. At about 12 inches from the patient the surgeon holds in front of his nose a green disc, 3 mm. in diameter, and asks the patient to name the colour of the disc. The usual answer in such cases is "dirty white." Then while the disc is moved first to the inner and afterwards to the outer side—the patient meanwhile still directing his eye to the surgeon's nose—the question as to the colour is repeated. The name of the colour is then correctly given. A red disc of similar size is next taken, and the same procedure followed. By this means the relative central scotoma for red and green is made manifest. The other is afterwards similarly examined. A scotoma is said to be relative when colour-perception is diminished, while the light sense remains. It is said to be absolute when both light and colour perception have gone, thus creating a gap in the field of vision. In advanced cases of tobacco amblyopia this result is apt to occur, in which case the patient's central vision is destroyed, leaving him only the peripheral part of his field for visual purposes. The loss of central vision means incapacity to do any fine work.

What is the pathology of these cases of tobacco amblyopia? According to Samelsohn the changes present are due to a chronic interstitial inflammation of the papillo-macular bundle of fibres of the optic nerve, immediately behind the disc. Hence these cases have been described as belonging to the category of those included under the term of chronic retro-ocular neuritis. Most commonly, however, the term "retro-bulbar" is used in this connection. Why the eye should be spoken of as a "bulb," I cannot conceive. Possibly those who speak of "ophthalmia neonatorum" when they mean purulent conjunctivitis, may be able to throw some light upon this terminological inexactitude. But why, and how, does excessive smoking cause this toxæmic amblyopia? The answer to these questions involves the discussion of many interesting details. There is first the incidence of age. In young persons the disease is practically unknown: it is only during middle life that the cases are generally met with. So it has been said that probably the resistance to the effects of nicotine diminishes with age. Frequently in such patients there is a long history of excessive smoking. In this regard it is common for patients to express surprise on being informed that their loss of vision was due to their tobacco habit. "Why, I have smoked," they may say, "the same quantity of tobacco every day for twenty years, and have never been any the worse. Why should it affect me now?"

The query is one which is well worthy of consideration. In every case of smoking, and especially in chewing, tobacco, a certain amount of nicotine is swallowed with the saliva. According to Sir Lauder Brunton, "the substances which are produced by the dry distillation which the tobacco undergoes in the process of smoking are chiefly the volatile alkaloids, pyridine, picoline, and collidine, with other bases nearly resembling them in character, all of which are closely allied both in smell and physiological action to nicotine." The effect of swallowing such poisonous products, whatever their form, is ultimately to cause irritation of the mucous coat of the stomach, and consequently a chronic gastritis, as is shown by the furred condition of the tongue and the loss of appetite to which reference has been made. This chronic gastritis either interferes with the elimination of the tobacco alkaloids, or in combination with those alkaloids it creates a new toxin, more poisonous in its effects than the tobacco bases themselves. We possess, however, no certain knowledge as to the pathology of the disease, or as to the exact tissue changes upon which the amblyopia depends. By some it is thought that the central amblyopia is due to functional deficiency of the retina, increased by a toxic process. By others that the amblyopia is the expression not of disease of the papillo-macular bundle of fibres, but of the macula lutea, by which degeneration of its cells is caused. Whatever may be, however, the precise origin of the amblyopia and the nature of the nerve changes associated with it, there can nevertheless be very little doubt that the primary and essential condition to the evolution of these toxic symptoms is the chronic gastritis which follows excessive indulgence in the tobacco habit.

Another factor in the causation of these cases is the quality of the tobacco which is used. Excessive smoking sufficient to cause a toxic amblyopia is impossible with mild forms of tobacco. For certain safeguards exist by which this excess is prevented. For example, excessive indulgence in cigarette smoking is checked by the chronic pharyngitis which generally ensues as a consequence. Excessive smoking of a mild tobacco in pipes is prevented by the soreness of the tongue which is thus induced. Consequently inveterate smokers avoid mild forms of tobacco, by which these results are caused, and limit their smoking to tobacco of strong and powerful flavour, such as among workmen is represented by shag. With strong tobacco like shag smoking to excess can be indulged in without causing any local disturbing effects. But while the quality of the tobacco is usually the same, the quantity used varies. Among workmen the minimum amount constituting the excess appears to be a quarter of an ounce daily. Frequently this amount is exceeded, but I have never come across a case in which the quantity has been less.

An interesting symptom commonly associated with tobacco amblyopia is hemeralopia or day blindness. Be careful not to confound this term with nyctalopia—night blindness. The confusion caused by German and American writers using these terms in exact contradiction has led ophthalmic surgeons in this country to employ the English terms of day and night blindness, to the exclusion of the scientific equivalents. Patients with tobacco amblyopia often assert that their vision is much worse in a bright light than when the light is dull. A case is recorded by Fuchs of the coachman of a medical man who was able to distinguish the numbers on the doors of houses in the dusk, while he was unable to do so during the day. This day-blindness is probably dependent upon the strong light causing contraction of the pupils by which the defective central vision is brought more into play; while in a dull light, with more dilatation of the pupil, the peripheral part of the field of vision is made available for visual purposes.

We come lastly to the all-important question of the treatment of these cases. This resolves itself into two main features: (1) the absolute prohibition of every form of the tobacco habit; and (2) the administration of suitable remedies to cure the chronic gastritis. The first is always a difficult matter for the patient. Having for years been a slave to smoking, the announcement that from henceforth he must cease being so comes to him as a shock. Nevertheless, he is generally wise enough to appreciate the necessity of following the advice given in this regard, especially when the alternative is pointed out that permanent loss of vision, in more or less degree, must otherwise eventually ensue. Occasionally, however, a patient may disregard the necessary prohibition, or seek to evade it. In one such case I was much puzzled, for despite the fact that the man asserted his absolute cessation of smoking, there was no improvement in his symptoms after a month's treatment. After questioning him again closely, he shamefacedly admitted that although he had ceased to smoke, he had taken to chewing tobacco as an alternative. I reminded him of his folly, and I suppose that he chose to continue it, for I did not see him again. I generally find that a mixture of iron and strychnine yields the best result, so far as internal medication is concerned. In the course of a month or so an improvement in the patient's general health becomes apparent, and with this is associated some recovery of the vision. The treatment, however, should be continued for at least twelve months, the progress being usually very gradual, and this after some months may entirely cease, leaving the vision permanently impaired. But, save in the neglected cases the prognosis may be regarded as good. Any return to the smoking habit after the cessation of the treatment should always at first be of a tentative nature in order that the patient may safeguard himself against a relapse.

Next week's Clinic will be on "Fibroid Lung," by A. J. Jex-Blake.

